# Association of a CT-Based Clinical and Radiomics Score of Non-Small Cell Lung Cancer (NSCLC) with Lymph Node Status and Overall Survival

**DOI:** 10.3390/cancers12061432

**Published:** 2020-05-31

**Authors:** Francesca Botta, Sara Raimondi, Lisa Rinaldi, Federica Bellerba, Federica Corso, Vincenzo Bagnardi, Daniela Origgi, Rocco Minelli, Giovanna Pitoni, Francesco Petrella, Lorenzo Spaggiari, Alessio G. Morganti, Filippo Del Grande, Massimo Bellomi, Stefania Rizzo

**Affiliations:** 1 Medical Physics, IEO European Institute of Oncology IRCCS, via Ripamonti 435, 20141 Milan, Italy; Francesca.botta@ieo.it (F.B.); lisa.rinaldi@ieo.it (L.R.); daniela.origgi@ieo.it (D.O.); 2 Molecular and Pharmaco-Epidemiology Unit, Department of Experimental Oncology, IEO European Institute of Oncology IRCCS, via Ripamonti 435, 20141 Milan, Italy; Federica.bellerba@ieo.it (F.B.); Federica.corso@ieo.it (F.C.); 3 Department of Statistics and Quantitative Methods, University of Milano-Bicocca, 20126 Milan, Italy; vincenzo.bagnardi@unimib.it; 4 Post-graduate School in Radiodiagnostics, Università degli Studi di Milano, Via Festa del Perdono 7, 20122 Milan, Italy; rocco.minelli@unimi.it (R.M.); mariagiovanna.pitoni@unimi.it (G.P.); 5 Department of Thoracic Surgery, IEO European Institute of Oncology IRCCS, via Ripamonti 435, 20141 Milan, Italy; Francesco.petrella@ieo.it (F.P.); lorenzo.spaggiari@ieo.it (L.S.); 6 Department of Oncology and Hemato-Oncology, Università degli Studi di Milano, 20122 Milan, Italy; Massimo.bellomi@ieo.it; 7 Department of Experimental, Diagnostic and Specialty Medicine—DIMES, University of Bologna, 40126 Bologna, Italy; Alessio.morganti2@unibo.it (A.G.M.); stefania.rizzo@eoc.ch (S.R.); 8 Clinica di Radiologia EOC, Istituto Imaging della Svizzera Italiana (IIMSI), Via Tesserete 46, 6900 Lugano, Switzerland; filippo.delgrande@eoc.ch; 9 Department of Radiology, IEO European Institute of Oncology IRCCS, via Ripamonti 435, 20141 Milan, Italy

**Keywords:** computed tomography, lung cancer, lymph nodes, radiomics, overall survival, reconstruction algorithms

## Abstract

Background: To evaluate whether a model based on radiomic and clinical features may be associated with lymph node (LN) status and overall survival (OS) in lung cancer (LC) patients; to evaluate whether CT reconstruction algorithms may influence the model performance. Methods: patients operated on for LC with a pathological stage up to T3N1 were retrospectively selected and divided into training and validation sets. For the prediction of positive LNs and OS, the Least Absolute Shrinkage and Selection Operator (LASSO) logistic regression model was used; univariable and multivariable logistic regression analysis assessed the association of clinical-radiomic variables and endpoints. All tests were repeated after dividing the groups according to the CT reconstruction algorithm. *p*-values < 0.05 were considered significant. Results: 270 patients were included and divided into training (n = 180) and validation sets (n = 90). Transfissural extension was significantly associated with positive LNs. For OS prediction, high- and low-risk groups were different according to the radiomics score, also after dividing the two groups according to reconstruction algorithms. Conclusions: a combined clinical–radiomics model was not superior to a single clinical or single radiomics model to predict positive LNs. A radiomics model was able to separate high-risk and low-risk patients for OS; CTs reconstructed with Iterative Reconstructions (IR) algorithm showed the best model performance.

## 1. Introduction

The lung represents the second most frequent site of cancer each year, and the first cause of death from cancer [1]. For early stages, when surgery is the primary option, complete lymph node (LN) excision with a microscopic evaluation is the most accurate method for determining LN metastasis. However, the best local control and decreased risk of residual lesions guaranteed by the dissection of a high number of LNs is associated with greater trauma for patients, such as prolonged air leaks, excessive chest tube drainage and prolonged hospitalization [2,3,4]. A method that could non-invasively predict the presence of LN metastasis would therefore be helpful to guide systematic dissection and might be considered, for example, in patients with small tumours and no apparent enlarged LNs [5], or in the presence of many co-morbidities.

Radiomics is an emerging translational field of research, aiming to extract mineable high-dimensional data from clinical images, containing information that may reflect the underlying patho-physiology of a tissue [6]. Many recent studies have evaluated the associations between radiomics and the prognosis of various tumours, such as lung adenocarcinoma [7,8,9], renal cancer [10], hepatocellular carcinoma [11,12], glioblastoma [13], and ovarian cancer [14].

Some methodological issues have been raised regarding radiomics studies, including the possibility that differences in quantitative data may depend on different parameters of acquisition and reconstruction [15]. It has also been widely demonstrated that at least internal, possibly external, and independent validation of the model is always needed [16].

The purpose of this study was to evaluate whether a model based on quantitative CT radiomic and clinical features of lung cancer patients may be associated with LN status and with overall survival (OS); a secondary purpose was to assess the influence of CT reconstruction algorithms on the quantitative parameters and on the performance of the predictive models.

## 2. Results

### 2.1. Patients

All patients underwent anatomical resection with radical lymphadenectomy; no cases of intraoperative nodal upstaging to pN2 from cN0 or cN1 were observed.

A total of 270 patients were enrolled and randomly divided into training (n = 180) and validation datasets (n = 90). The histological type was adenocarcinoma or squamous cell carcinoma in 254/270 patients; other types were present in 16/270 patients. The distribution of histological type between the training set and the validation set did not show significant differences (*p* = 0.16). Other baseline characteristics of the cohort are summarized in Table 1.

The clinical and radiological parameters are comparable between the two datasets, although a slightly higher percentage of patients with pT > 1 was randomly allocated in the validation set (standardized mean difference = 0.21).

A total of 881 radiomic features was calculated for each patient. The numbers of radiomic features which survived after elimination of identical features and selected as the most stable and reproducible after phantom analysis and Analysis of Variance (ANOVA) are reported in Figure S1 for the different cases (analysis performed on the entire Filtered Back Projection (FBP) + Iterative Reconstructions (IR) dataset and analysis performed separating the patients according to FBP or IR reconstructing algorithm).

#### 2.1.1. Lymph Nodes

##### Analysis on the Entire Dataset (FBP + IR)

**Figure 1:**
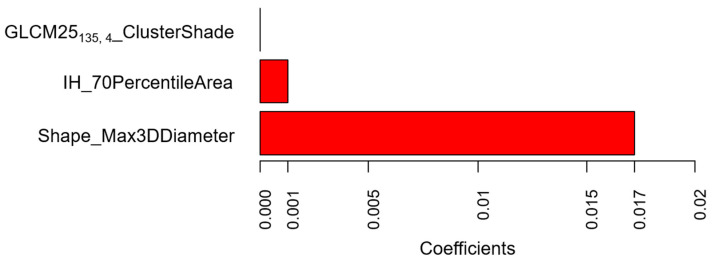
Values of the coefficients of the Least Absolute Shrinkage and Selection Operator (LASSO) logistic regression model for the prediction of positive lymph nodes according to radiomic features (training set). The plot shows the model coefficients of the three radiomic features selected as significantly associated with lymph node status. These coefficients were used to calculate the radiomics score used to predict lymph node status in the validation set.

The radiomics score obtained for the prediction of positive lymph nodes (LNs) on the entire training dataset consisted of three radiomic features: ClusterShade from GLCM25 category calculated along 135° direction with four voxels offset (GLCM25_135, 4__CIusterShade), 70th percentile of the intensity values in the cumulative histogram (IH_70PercentileArea), and the maximum diameter evaluated on the 3D lesion volume (Shape_Max3DDiameter). Coefficients of the Least Absolute Shrinkage and Selection Operator (LASSO) logistic regression model for the calculation of the radiomics score are shown in Figure 1.

Among clinical variables, univariable analysis in the training set showed that mixed site (meaning that the tumour showed a transfissural growth into two lobes) and nodule size (the larger, the worse) were significantly associated with positive LNs. Multivariable analysis confirmed the importance of the site (Table 2).

**Figure 2:**
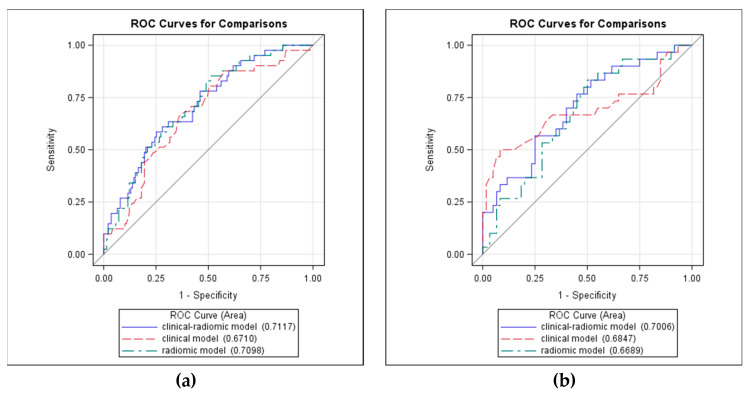
ROC curves for prediction of positive lymph nodes in (**a**) the training set and (**b**) the validation set according to clinical, radiomics and clinical–radiomics models. The plots show the ROC curves of the three models and the associated values of the Area under the Curves (AUC).

Receiving Operating Characteristic (ROC) curves for prediction of positive LNs showed no significant differences in performance of the combined clinical–radiomics model, as compared to a single radiomics or single clinical model in the training and validation sets (Figure 2).

##### Analysis on Datasets Separated According to FBP or IR Algorithm

**Figure 3:**
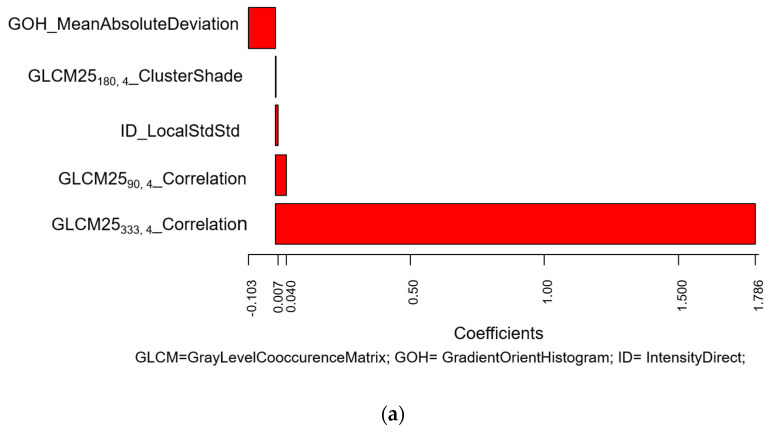

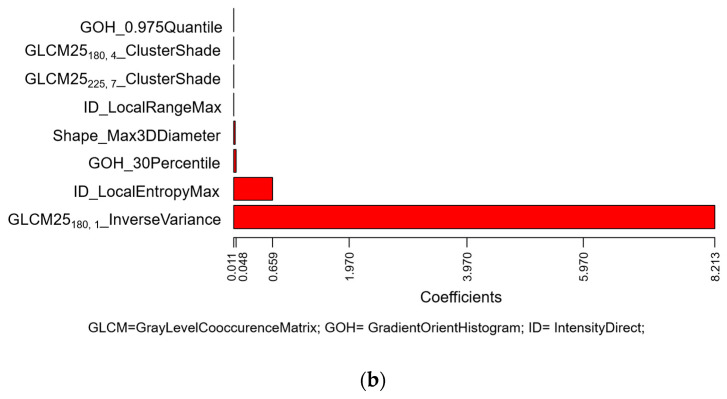
Values of the coefficients of the LASSO logistic regression model for the prediction of positive lymph nodes according to radiomic features for (**a**) FBP algorithm and (**b**) IR algorithm (training set). The plots show the model coefficients of the (**a**) five and (**b**) eight radiomic features selected as significantly associated with lymph nodes in the two subsets of patients with (**a**) FBP algorithm and (**b**) IR algorithm. These coefficients were used to calculate the corresponding radiomic scores used to predict lymph node status in the validation set for the two separate sub-samples.

Figure 3 shows coefficients of the LASSO logistic regression model for the radiomics score prediction of positive LNs, separately for FBP and IR (training set).

In both cases, a prevalent role of the textural features is evident, whereas this was not observed when considering the entire dataset including both FBP and IR. ROC curves in the training and validation sets acquired with FBP and IR were separately evaluated (Figure S2).

The overall performance of the combined model to predict LN positivity was higher for IR than FBP algorithms, with an Area Under the Curve (AUC) of 0.76 for IR compared to 0.61 for FBP in the validation set.

#### 2.1.2. Overall Survival

##### Radiomic Analysis on the Entire Dataset (FBP + IR)

**Figure 4:**
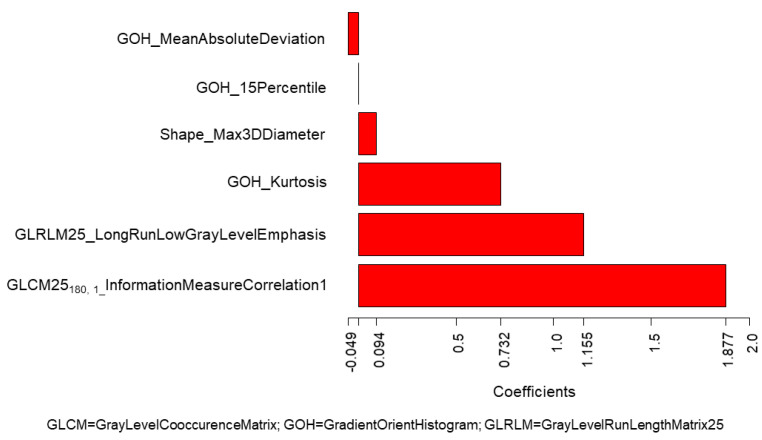
Values of the coefficients for Cox regression LASSO model for prediction of overall survival according to radiomic features (training set). The plot shows the model coefficients of the six radiomic features selected as significantly associated with overall survival. These coefficients were used to calculate the radiomic score used to predict overall survival in the validation set.

Looking at OS prediction, in the entire training set (including both FBP and IR), the Cox regression LASSO model selected six radiomic features for the radiomics score (Figure 4).

Table 3 shows parameters associated with OS in high-risk and low-risk patients, defined according to the third quartile of the radiomics score.

**Figure 5:**
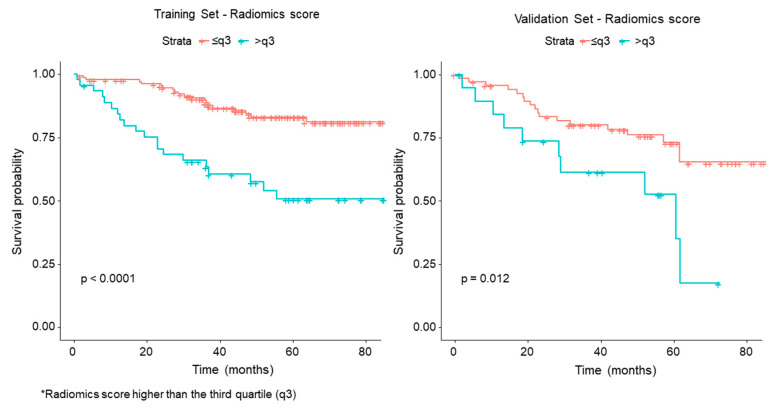
Kaplan–Meier curves and Log–Rank test for high-* and low-risk groups according to the radiomics score (* Radiomics score higher than the third quartile (q3)).

The percentage of deaths at the second and third years in the validation set for high-risk patients was almost double that of the low-risk patients (respectively, 35% vs. 19% and 45% vs. 23%). As shown in Figure 5, a significant difference in OS for the high- and low-risk groups according to the radiomics score was observed in the training set and confirmed in the validation set.

##### Radiomic Analysis on Datasets Separated According to FBP or IR Algorithm

**Figure 6:**
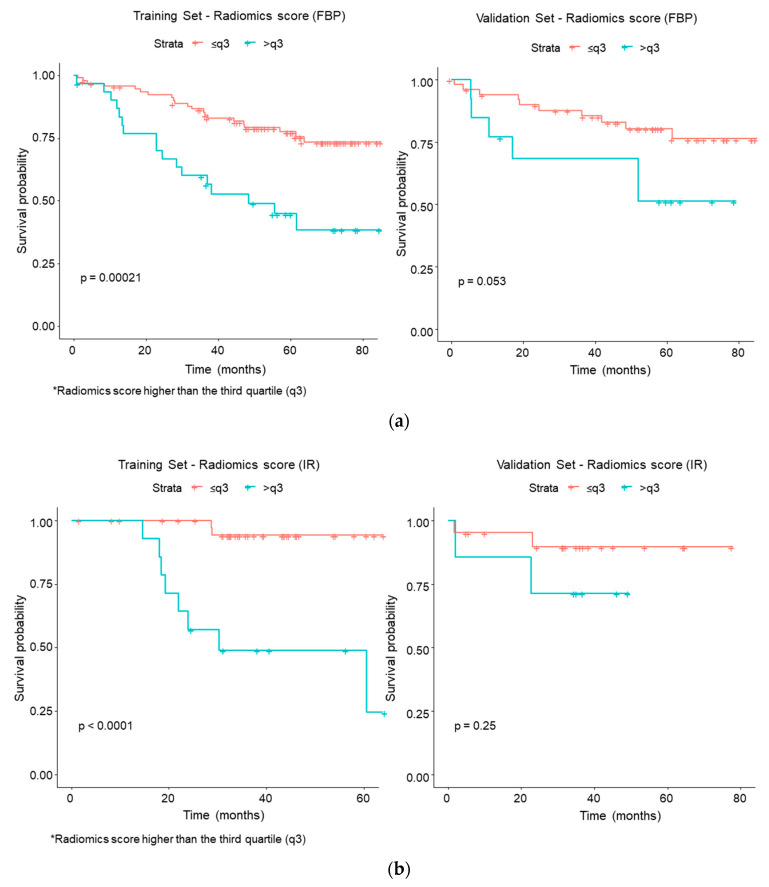
Kaplan–Meier curves and Log–Rank test for high * and low-risk groups according to the radiomics score for (**a**) FBP algorithm and (**b**) IR algorithm (* Radiomics score higher than the third quartile (q3)).

The above-described results were further confirmed after dividing the two groups according to the reconstruction algorithms (Figure 6).

##### Radiomic and Clinical Analysis

Clinical variables associated with OS in the model including both FBP and IR algorithms were tumour side, site and pT—tumour site and pT were associated with OS for the subgroup of patients with FBP algorithm, while only tumour site was associated with OS for the subgroup of patients with IR algorithm.

Table 4 summarizes the performance of the models including radiomic only, clinical only, and clinical–radiomic parameters, on both the entire FBP+IR datasets and on datasets separated according to reconstruction algorithm.

The best model performance for prediction of OS was obtained with the combined clinical–radiomic model for CT reconstructed with the IR algorithm.

## 3. Discussion

In this series, selected clinical and radiomic features showed association with the positivity of LN, although the combined radiomics–clinical model did not perform better than a single clinical or radiomics model. Among the clinical features, the mixed site was significant in the univariate and multivariate analysis. The importance of this feature is recognized by its role in Tumor-Nodes-Metastasis (TNM) staging, where the invasion of the visceral pleura makes the tumour belong to the T2 category, despite its size [17]. This result is concordant also with Li et al., who demonstrated a significant association between adjacent lobe invasion through pleural fissure and LN positivity [18]. The size of the tumour, measured on an axial image, was borderline-significant in the univariable and multivariable analysis. Size is a well-known feature for prognosis, as demonstrated by the sub-division of tumours in the T category according to size [17]. Its importance is confirmed in our series by the better performance of the single clinical model, after the inclusion of size as a feature. Likewise, the importance of size as a prognostic factor for LN positivity is demonstrated by one radiomic feature included in the score, the Shape_Max3DDiameter. This feature represents a measure of the maximum dimension of the lesion, evaluated in 3D, not directly related to the volume and is more precise than the maximum axial diameter. This may also account for the slightly better performance of the radiomics model compared to the clinical model, where the Max3DDiameter radiomic feature may provide a more precise definition of size than the maximum axial diameter. Other significant features in the radiomics score for LN positivity prediction were IH_70PercentileArea and GLCM25_135, 4__ClusterShadeClShade. IH_70PercentileArea belongs to the Intensity Histogram category and roughly indicates that the mean Hounsfield Units (HU) number within the lesion is associated with LN status. GLCM25_135, 4__ClusterShade, belonging to the GLCM category, is a measure of the global skewness. When the Cluster Shade parameter is high, the distribution of HU values is asymmetric. In our series, low values of this feature, that may be associated with intratumoral necrosis, were more frequently associated with positive LNs, whereas high values, encountered in lesions with calcifications and ground-glass opacities, were associated with negative LNs.

Despite the abovementioned associations, the combined clinical and radiomics model did not perform better than a single clinical or radiomics model. This result is discordant with Tan et al., who demonstrated that, in patients with resectable oesophageal carcinoma, a radiomics nomogram provided a good risk estimation of LN metastasis and outperformed size criteria [19]. However, unlike these authors, we did not evaluate LN radiomic features, because our purpose was to predict the presence of positive LNs according to the characteristics of the primary tumour. Conversely, Yang et al. [20] demonstrated a good performance of a radiomics-based nomogram extracted from lung tumour to predict LN metastases. Although we used a similar method, by performing the radiomics analysis on the lung tumour volume, our results may be different because Yang et al. included patients with CT examinations acquired with the same parameters and reconstructed with the same algorithm [20]. In this regard, our entire sample is less homogeneous, because we included CTs obtained from different scanners and reconstructed with two different algorithms. However, it must be pointed out that from a methodological point of view, we tried to take this into account by performing a preliminary selection with ANOVA to choose features that were not significantly affected by the use of different scanners or reconstruction algorithms. This is important because reproducibility and differences in acquisitions and reconstructions are frequent issues in retrospective studies, which currently represent the majority of radiomics studies.

As a consequence, the poor performance of our predictive model, including radiomic and clinical parameters, as compared to the model based on clinical variables alone, could be due to the fact that feature selection with ANOVA may have eliminated features which potentially carry relevant predictive information, but are significantly affected by the scanner or reconstruction algorithm. It is possible that, in a more homogeneous dataset, such features would survive the feature selection process and would enter the predictive model with a significant improvement in terms of performance.

In order to verify this hypothesis, we performed a separate analysis, according to reconstruction algorithms: FBP, the most common analytical reconstruction method, which uses a 1D filter on the projection data before back-projecting them onto the image space [20,21], and IR, which optimizes the reconstruction with multiple iterations of forward and back projection between image and projection space. With the advances in computing technology, IR has progressively replaced FBP in routine CT practice because it makes it possible to reduce artefacts [22]. Therefore, while retrospective databases are likely to include CT images reconstructed with either FBP or IR, future studies will mainly involve IR, unless FBP is proved to be more indicated for quantitative analysis and expressly added to the clinical IR reconstruction. In this series, for association with LN status, the radiomics score associated with FBP included five quantitative features, where the most important coefficients were attributed to two different features of Correlation type, belonging to the GLCM25 category. Correlation features measure the linear dependency of voxel intensities at each point with those of neighbouring pixels at fixed distances along different directions. In our series, this feature was high for lung tumours with air bronchograms and with high heterogeneity due to contrast-enhancement (particularly in the presence of subtle vascularization within the tumour). For CT examinations reconstructed with IR, the most important coefficients were attributed to the InverseVariance feature from the GLCM2.5 category, calculated along 180° direction with one voxel offset, and to the LocalEntropyMax feature from ID category. The larger the changes in grey values, the higher the GLCM contrast; the lower the values, corresponding to an inhomogeneous texture, the higher the association with LN positivity. LocalEntropyMax (Maximum of Local Entropy) is a primary-order statistics feature measuring the entropy within the Region of Interest (ROI), and shows high values even for small areas of entropy within a single voxel. Higher local entropies were associated with LN positivity. These results confirmed that focusing on a more homogeneous dataset (as far as CT reconstruction algorithm is concerned) may make it possible to identify texture features significantly associated with LN status, not revealed in a heterogeneous dataset. We were still not able to demonstrate that, in a homogeneous dataset, the performance of a combined clinical–radiomics model is superior to a model based on clinical or radiomic data only, probably due to the fact that dividing the dataset according to a CT reconstruction algorithm considerably reduced the size of each training and validation set. Nonetheless, promising results were found in the IR dataset (Figure S2), which may deserve to be further investigated on a larger sample.

The analysis of association between clinical and radiomic features with OS showed a very good performance of the radiomics score, that significantly separated patients into high-risk and low-risk groups (*p* < 0.0001 in the training set; *p* = 0.012 in the validation set). Among radiomic features, GLCM25_180, 1__InformationMeasureCorrelation1, GLRLM25_LongRunLowGrayLevel Emphasis, GOH_Kurtosis and Shape_Max 3DDiameter were included in the score. GLCM25_180, 1__InformationMeasureCorrelation1 belongs to the GLCM category and quantifies the degree of randomness within the tumour, in terms of entropy and statistical disorder. GLRLM25_LongRunLowGrayLevel Emphasis belongs to the GLRLM category, and quantifies runs, intended as consecutive pixels with the same grey level. GOH_Kurtosis indicates the flatness of the curve of values, without concern for spatial relationships. A prevalent direction for the voxel intensity gradient indicates that structures within the 3D volume of interest (VOI) develop along a precise direction (e.g., an intratumoral vessel). Shape_Max 3DDiameter (see features of the radiomics score for LN) was associated with OS, with high values (large lesions) associated with worse OS.

This study has some limitations. The number of patients with malignant lymph nodes (pN1) in our group was relatively small (71/270; 26%) and this may account for different results compared to previous papers. However, the inclusion of patients with pN2 introduced a bias related to the neo-adjuvant chemotherapy. The CT examinations included in this study were performed over quite a long period of time (four years), and this may have affected the acquisition protocols. However, we specifically performed a preliminary ANOVA test to select stable and reproducible radiomic features. Furthermore, since the most important change in CT acquisition during the period selected was the reconstruction algorithm, we performed a separate analysis according to the algorithm used, as described. These subgroup analyses were performed on even smaller samples, which may have generated incidental results; they could therefore be considered as exploratory analyses that need to be validated in future, larger studies. Finally, segmentation was carried out by a single operator. Although this has been considered a limitation in previous studies [23], the inclusion of multiple slices to obtain a volume, instead of a single ROI, as well as the methods of extraction of the features by dedicated software may overcome this limitation.

## 4. Materials and Methods

Patient selection. The Institutional Review Board approved this retrospective study (UID 2172), waiving the need for informed consent. The study population was retrospectively selected from a database of patients with lung cancer staged up to T3 N1, operated on between 01/01/2012 and 01/08/2016. Preoperative staging was performed by whole body CT and Fluorodeoxyglucose Positron Emission Tomography (FDG PET) scan; in the event of suspected cN2 disease, preoperative endobronchial ultrasound trans bronchial needle aspiration (EBUS TBNA) was performed to rule out or to confirm lymph node involvement. Inclusion criteria were: availability of pre-surgical CT at our Institution after contrast medium injection, with helical mode, 120 kVp, 2.5 mm slice thickness, 2.5 mm spacing; reconstruction performed with “Body” filter and “Standard” convolution kernel; surgery performed at our Institution; availability of histology, pathological node status (pN0; pN1), grading. Exclusion criteria were: CT performed with parameters different to those specified above; pre-operative chemotherapy.

CT imaging. Examinations were randomly performed on the following CT scanners: Lightspeed Ultra, Lightspeed 16; Optima 660; Discovery CT750 HD (all GE Healthcare, Milwaukee, WI, USA). All scans were acquired in the portal venous phase and segmentations were performed on that series. All scanners implemented current modulation; Light speed 16 and Light Speed Ultra were equipped only with longitudinal z-axis modulation, while Optima 660 and Discovery CT750 also had angular xy modulation.

Clinical and radiological data recording. The following data were recorded: age; gender; grading; side; site (upper, medium, lower, mixed); axial nodule size; pT; pN; CT scanner; CT reconstruction algorithm (Filtered Back Projection, FBP, or Iterative Reconstruction, IR); exposure (mAs, defined as mA_central slice * RevolutionTime * SliceThickness)/(Pitch*TotalCollimationWidth); contrast medium (Visipaque^®^ 320, GE Healthcare; Ultravist^®^ 370, Bayer; Iomeron 350, Bracco Imaging; Xenetix 350, Guerbet); status (alive or deceased); date of last contact or death.

Lesion segmentation: On each axial CT image including the lung nodule, a radiologist traced free-hand 2D regions of interest, resulting in a 3D volume of interest (VOI) that was converted into a DICOM RT Structure format (AW Server 2.0 workstation, GE Healthcare).

Radiomic feature extraction: CT images and VOI were imported into the IBEX V 1.0 β tool (Imaging Biomarker Explorer Software [24]) for the extraction of radiomic features. The “Resample_VoxelSize” pre-processing was used to resample images to the same pixel size, chosen as the value most frequently observed in the dataset (0.7 × 0.7 mm^2^, the values in patient images ranging from 0.59 × 0.59 mm^2^ to 0.98 × 0.98mm^2^). “Threshold_Image_MaskXF” pre-processing, that excludes voxels on the ROI edge having intensity outside a user-defined range, was used to exclude parenchyma voxels erroneously included during manual delineation (threshold: −400 Hounsfield Units). All the features available in IBEX for the following categories were extracted: Shape, Intensity Histogram (IH), Intensity Direct (ID), Grey Level Co-occurrence Matrix (GLCM) 2.5, Grey Level Run Length Matrix (GLRLM) 2.5, Neighbour Intensity Difference (NID) 2.5, and Gradient Orient Histogram (GOH). GLCM and GLRLM were calculated comparing voxel intensities along 8 different directions (0°, 45°, 90°, 135°, 180°, 225°, 270°, 315°) and with three different offsets between voxels (1, 4 and 7 voxels). The full list of features calculated by IBEX for each category is reported in [24], also including references for feature definition and formula. The [0–4096] HU interval was discretized in 256 bins for GLCM2.5 calculation, and 64 bins for the other categories.

### Statistical Analysis

Repeatability and reproducibility: In order to select the most stable and reproducible radiomic features, we first selected stable features with Overall Concordance Correlation Coefficient (OCCC) >0.95 based on the test-retest experiments on a phantom, where identical measures in different tests are expected. We then used one-way ANOVA to assess the features’ reproducibility according to contrast medium, scanner, reconstruction algorithm, and exposure. Features with significantly different means according to at least one of the abovementioned parameters were considered not robust and excluded.

Training and validation datasets: We randomly selected 2/3 of the patients as a training dataset, and 1/3 as a validation dataset.

Positive LN prediction: The Least Absolute Shrinkage and Selection Operator (LASSO) logistic regression model was used to select radiomic features associated with positive LNs. We combined selected features into a radiomics score. We assessed the predictive accuracy of the radiomics score for positive LNs by calculating the Area Under the Curve (AUC). The association of clinical variables (age, gender, side, site, and nodule size) with positive LNs was assessed with univariable and multivariable logistic regression analysis. A clinical score was obtained as a linear combination of the selected clinical variables weighted by their respective coefficients; the corresponding AUC was then calculated for both datasets. Finally, a radiomics–clinical score was obtained by applying a logistic regression multivariable model to the above-mentioned scores, and the corresponding AUC was calculated for both datasets. The AUC for the radiomics, clinical and clinical–radiomics models were compared with the DeLong test [25]. We replicated all the analyses separately on the two subgroups of patients with CT images reconstructed with FBP or IR algorithms. For each subgroup, AUCs for the radiomic, clinical and clinical–radiomics models were compared with the DeLong test [25].

OS prediction: Overall survival was calculated from the date of CT to the date of death or last follow-up, whichever occurred first. The LASSO Cox regression model was used to select the radiomic features predicting OS. We combined the selected features into a radiomics score as a linear combination of the selected features weighted by their respective coefficients. The association of the radiomics score with OS was assessed in the training and validation datasets by using Kaplan–Meier survival analysis. For this, the patients were classified into high-risk or low-risk groups according to the radiomics score, by using the third quartile as the threshold. The difference in the survival curves of the high-risk and low-risk groups was evaluated by using the Log–Rank test. The predictive accuracy of the radiomics score for OS was assessed in both datasets [26]. The association of clinical variables (age, gender, side, site, nodule size, histological type, grading, pT and pN) with OS was assessed with univariable and multivariable analysis. A clinical score was obtained as a linear combination of the clinical variables weighted by their respective coefficients. Finally, a combined radiomics–clinical score was obtained by applying a Cox regression multivariable model to the above-mentioned scores, and the corresponding C-index was calculated for the clinical–radiomics model in both datasets.

To assess the role of reconstruction algorithms on the models, we replicated all the analyses separately on the two subgroups, according to FBP and IR algorithms.

*p*-values < 0.05 were considered statistically significant. The analyses were performed using SAS software (SAS Institute Inc., Cary, USA), version 9.4 and R software (http://www.Rproject.org), version 3.5.3. (R Core Team, R Foundation for Statistical Computing, Vienna, Austria). More details are provided in the Supplementary Materials.

Training and validation datasets: The subjects were randomly split into training and validation groups so that 2/3rd of subjects were included in the training group and 1/3rd in the validation group. This allocation proportion is commonly used to ensure the model is trained on a sufficient number of patients, in order to obtain precise parameter estimates. From a previously published paper, this commonly used strategy was demonstrated to be close to optimal for reasonably sized datasets (*n* ≥ 100) with strong signals (i.e., 85% or greater full dataset accuracy) [27].

Positive LN prediction: The Least Absolute Shrinkage and Selection Operator (LASSO) logistic regression model was used to select radiomic features predicting positive LNs. First, a 10-fold cross-validation was computed to find the best lambda parameter. The regularization strength was selected as the minimum value that maximized the Area Under the receiver operating characteristics Curve (AUC). The R package “glmnet” was used to perform the LASSO logistic regression on the training dataset with standardized features. The coefficients were then returned to the original scale. We then combined the selected features into a radiomics score as a linear combination of the selected features weighted by their respective coefficients. We also assessed the predictive accuracy of the radiomics score for positive LNs both in the training and in the validation datasets by calculating the AUC. The association of clinical variables (age, gender, side, site, and nodule size) with positive LNs was assessed with univariable and multivariable logistic regression analysis. Multivariable analysis included the clinical variables with a *p*-value < 0.10 at univariable analysis. A clinical score was obtained as a linear combination of the selected clinical variables weighted by their respective coefficients, and the corresponding AUC was calculated for the clinical model for both the training and the validation datasets. Finally, a radiomics–clinical score for prediction of positive LNs was obtained by applying a logistic regression multivariable model to the radiomics score and the clinical score, and the corresponding AUC was calculated for the clinical–radiomics model for both the training and the validation datasets. The AUC for the radiomics, the clinical and the clinical–radiomics models were compared with the DeLong test [16].

Since the measurements of most radiomic features showed dependency according to the different CT reconstruction algorithms and were excluded from the previous analysis, we replicated all the above-mentioned analyses separately on the two groups of subjects, according to FBP and IR reconstruction algorithms including all the features that proved to be repeatable after test-retest analysis. A leave-one-out cross validation was performed for both algorithms and a LASSO logistic model was implemented with the same settings as previously described. For each subgroup of patients, AUC for the radiomics, clinical and clinical–radiomics models were compared with the DeLong test [16].

OS prediction: Overall survival was calculated from the date of CT to the date of death or last follow-up, whichever occurred first. We censored patients who had not died during the study period or until the date they were lost to follow-up, whichever occurred first, and we estimated their overall survival time. The LASSO Cox regression model was used to select the radiomic features that are most useful to predict OS. First a 10-fold cross-validation based on partial log-likelihood deviance was computed to find the best lambda parameter. The regularization strength was selected as the minimum value that minimizes the partial log-likelihood deviance. The R package “glmnet” was used to perform the LASSO Cox regression as previously described. We then combined the selected features into a radiomics score as a linear combination of the selected features weighted by their respective coefficients. The potential association of the radiomics score with OS was assessed in the training dataset and validated in the validation dataset by using Kaplan–Meier survival analysis. For this, the patients were classified into high-risk or low-risk groups according to the radiomics score, by using the third quartile as the threshold. The difference in the survival curves of the high-risk and low-risk groups was evaluated by using the Log–Rank test. The predictive accuracy of the radiomics score for OS was assessed in both datasets by calculating the Harrel concordance index (C-index) with 95% Confidence Intervals (CI) [17]. The association of clinical variables (age, gender, side, site, nodule size, histological type, grading, pT and pN) with OS was assessed with univariable and multivariable Cox regression analysis. Multivariable analysis included the clinical variables with a *p*-value < 0.10 at univariable analysis. A clinical score was then obtained as a linear combination of the selected clinical variables weighted by their respective coefficients and the corresponding C-index was calculated for the clinical model for both the training and the validation datasets. Finally, a radiomics–clinical score for prediction of OS was obtained by applying a Cox regression multivariable model to the radiomics score and clinical score, and the corresponding C-index was calculated for the clinical–radiomics model for both the training and the validation datasets.

As described for positive LN prediction, we replicated all the above analyses separately on the two groups of subjects with the FBP and IR reconstruction algorithm.

## 5. Conclusions

In conclusion, a combined clinical–radiomics model was not superior to a single clinical or radiomics model in predicting LN metastases in lung cancer patients, whereas a radiomics score was able to significantly separate high-risk and low-risk patients for OS.

For the prediction of OS, the combined clinical–radiomics model demonstrated the best model performance for CT reconstructed with IR.

Based on these results, in the near future, clinical prediction of OS might include a radiomics score for better precision; furthermore, in similar radiomics studies, the extraction of radiomic features should include CT reconstructed with IR.

## Figures and Tables

**Table 1:** Baseline characteristics of the study population.

Characteristic	All Patients (N = 270) N (%)	Training Set (N = 180) N (%)	Validation Set (N = 90) N (%)
Age (years) ^	67.4 (61.0–72.6)	66.6 (60.7–72.2)	68.4 (62.3–72.8)
Gender			
Female	103 (38%)	74 (41%)	29 (32%)
Male	167 (62%)	106 (59%)	61 (68%)
Grading			
1	30 (13%)	21 (14%)	9 (12%)
2	82 (36%)	56 (37%)	26 (33%)
3	117 (51%)	74 (49%)	43 (55%)
Missing	41	29	12
Side			
Right	153 (57%)	102 (57%)	51 (57%)
Left	117 (43%)	78 (43%)	39 (43%)
Site			
Upper	154 (57%)	101 (56%)	53 (59%)
Medium	12 (4%)	9 (5%)	3 (3%)
Lower	93 (34%)	65 (36%)	28 (31%)
Mixed	11 (4%)	5 (3%)	6 (7%)
Nodule size (mm) ^	31 (18–45)	28 (17–45)	36 (22–46)
pT			
0	3 (1%)	1 (1%)	2 (2%)
1	97 (36%)	74 (41%)	23 (26%)
2	124 (46%)	76 (42%)	48 (53%)
3	46 (17%)	29 (16%)	17 (19%)
pN			
pN0	199 (74%)	139 (77%)	60 (67%)
pN1	71 (26%)	41 (23%)	30 (33%)
Algorithm type			
FBP	187 (69%)	130 (72%)	57 (63%)
IR	83 (31%)	50 (28%)	33 (37%)
Status			
Alive	202 (75%)	140 (78%)	62 (69%)
Deceased	68 (25%)	40 (22%)	28 (31%)
Follow-up (months) ^	46.1 (29.8–63.3)	47.0 (32.0–65.2)	45.5 (22.7–59.6)

FBP = Filtered Back Projection; IR = Iterative Reconstructions; ^ Median (InterQuantile Range).

**Table 2:** Univariable and multivariable Odds Ratios for the association between clinical variables with positive lymph nodes (training set).

Variable	Univariable Analysis		Multivariable Analysis *	
	OR (95%CI)	*p*-value	OR (95%CI)	*p*-value
Age (years)	1.02 (0.98–1.06)	0.38	-	-
Gender (females vs males)	0.78 (0.38–1.61)	0.50	-	-
Side (left vs right)	0.61 (0.29–1.26)	0.18	-	-
Site				
Medium vs. Upper	0.42 (0.05–3.67)	0.14	0.35 (0.04–3.20)	0.13
Lower vs. Upper	0.85 (0.39–1.82)	0.22	0.79 (0.36–1.72)	0.27
Mixed vs. Upper	**13.57 (1.44–127.43)**	**0.01**	**10.94 (1.13–105.40)**	**0.02**
Nodule size (mm)	1.01 (1.00–1.02)	0.07	1.01 (1.00–1.02)	0.12

CI = Confidence Intervals; OR = Odds Ratio; * includes radiomic score and clinical variables significantly associated with lymph nodal status at univariable analysis (*p* < 0.10). Significant values in bold.

**Table 3:** Overall Survival in high-risk and low-risk patients according to the radiomics score.

	Training Data Set			Validation Data Set		
	High-Risk Group *	Low-Risk Group	Total	High-Risk Group *	Low-Risk Group	Total
**No. of patients**	45 (25%)	135 (75%)	180 (100%)	20 (22%)	70 (78%)	90 (100%)
**Follow-up time**						
Median (IQR)	37.0 (19.3-62.2)	49.0 (35.5–68.4)	47.0 (32.6–66.1)	33.0 (17.2–56.4)	47.0 (28.9–61.6)	46.0 (23.6-60.6)
Shortest (months)	0.8	0.9	0.8	1.6	0.2	0.2
**No. of events**						
At 1 year	8 (18%)	3 (2%)	11 (6%)	3 (15%)	3 (4%)	6 (7%)
At 3 years	17 (38%)	14 (10%)	31 (17%)	7 (35%)	13 (19%)	20 (22%)
At 5 years	20 (44%)	19 (13%)	39 (22%)	9 (45%)	16 (23%)	25 (29%)

* Radiomics score higher than the third quartile.

**Table 4:** Model performance statistics.

Model	Algorithm	C-Index (95%CI) Training Set	C-Index (95%CI) Validation Set
Radiomic	FBP + IR	0.73 (0.66–0.80)	0.59 (0.47–0.70)
Clinical (side, site, pT)	FBP + IR	0.78 (0.71–0.85)	0.53 (0.41–0.65)
Clinical–radiomic	FBP + IR	0.77 (0.69–0.84)	0.57 (0.46–0.69)
Radiomic	FBP	0.69 (0.61–0.77)	0.61 (0.46–0.76)
Clinical (site, pT)	FBP	0.66 (0.57–0.75)	0.57 (0.43–0.72)
Clinical–radiomic	FBP	0.70 (0.63–0.78)	0.61 (0.46–0.75)
Radiomic	IR	0.82 (0.66–0.98)	0.82 (0.53–1.00)
Clinical (site)	IR	0.67 (0.50–0.85)	0.65 (0.38–0.91)
Clinical–radiomic	IR	0.84 (0.68–1.00)	0.89 (0.60–1.00)

## Supplementary Materials

The following are available online at https://www.mdpi.com/2072-6694/12/6/1432/s1, Figure S1: Number of radiomic features selected as the most reproducible, robust and reliable, Figure S2: ROC curves for prediction of positive lymph nodes in (a) the training set with FBP algorithm; (b) the validation set with FBP algorithm; (c) the training set with IR algorithm; (d) the validation set with IR algorithm according to clinical *, radiomics and clinical–radiomics models ^. (* clinical model includes site and nodule size as independent variables; ^ For the FBP algorithm: the clinical–radiomics model includes site, nodule size and radiomics score. For the IR algorithm: the clinical–radiomics model includes site and radiomics score. Nodule size was not included because of overlapping with the radiomic feature Shape_Max3DDiameter).
