# Supplementary material for: Association of a CT-Based Clinical and Radiomics Score of Non-Small Cell Lung Cancer (NSCLC) with Lymph Node Status and Overall Survival
Source: Cancers (Basel). 2020 May 31;12(6):1432. doi: 10.3390/cancers12061432 (PMC7352293; doi:10.3390/cancers12061432)
Supplement: Supplementary file 1 [file cancers-12-01432-s001.pdf]

cancers

MDPI

Association of a CT-Based Clinical and Radiomics Score of Non-Small Cell Lung Cancer (NSCLC) with Lymph Node Status and Overall Survival

Francesca Botta, Sara Raimondi, Lisa Rinaldi, Federica Bellerba, Federica Corso, Vincenzo Bagnardi, Daniela Origgi, Rocco Minelli, Giovanna Pitoni, Francesco Petrella, Lorenzo Spaggiari, Alessio G. Morganti, Filippo Del Grande, Massimo Bellomi and Stefania Rizzo

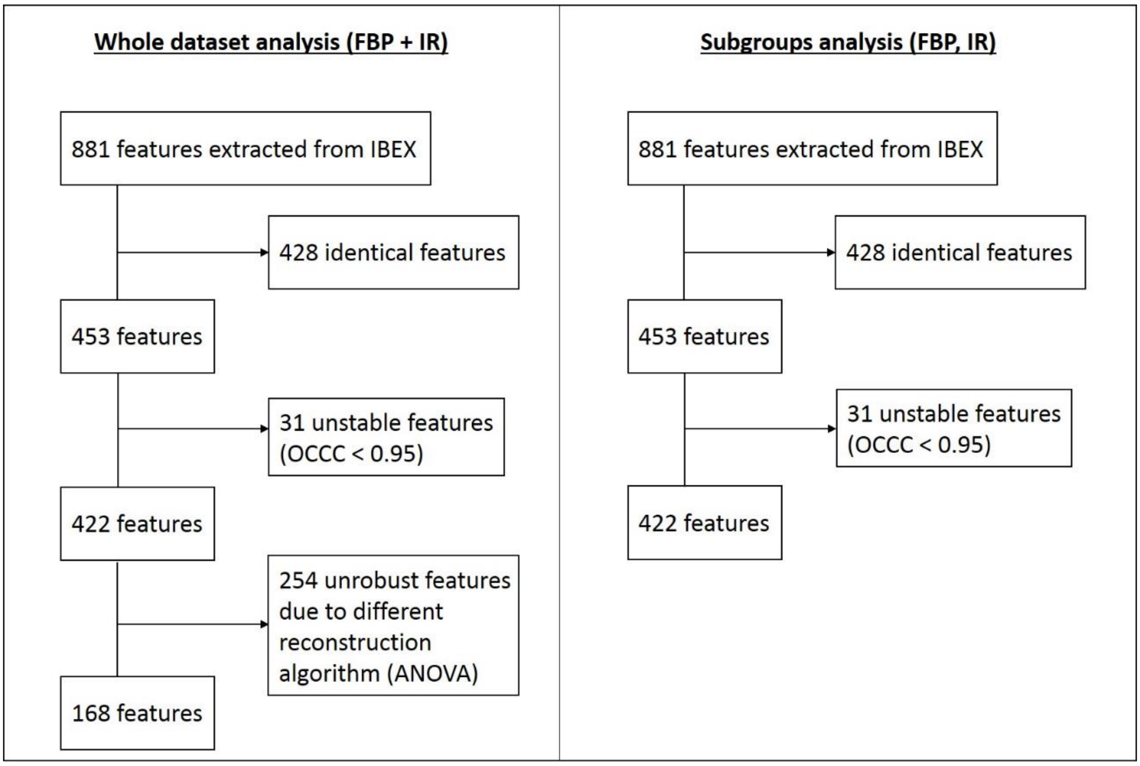

Figure S1. Number of radiomic features selected as the most reproducible, robust and reliable.

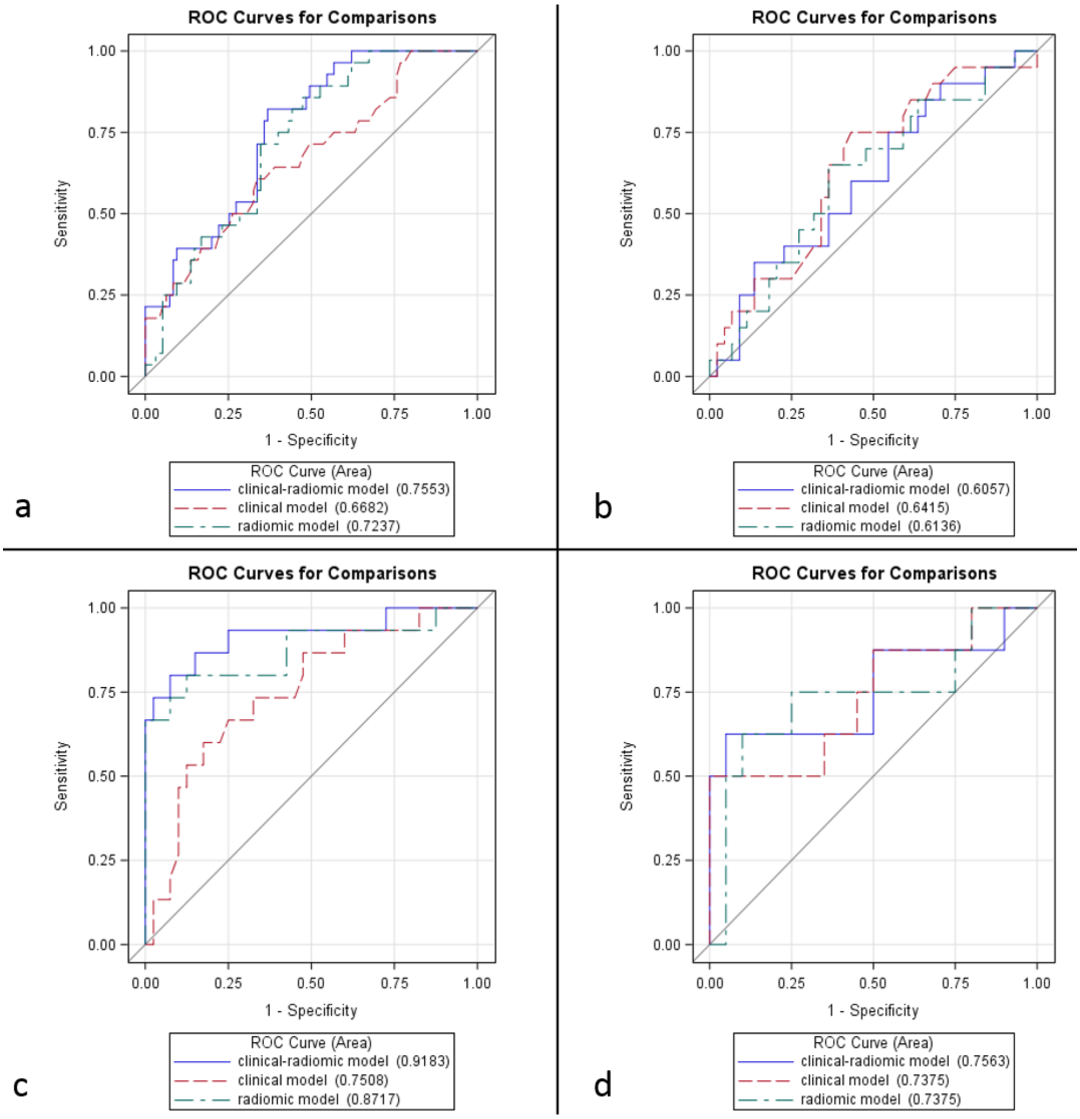

Figure S2. ROC curves for prediction of positive lymph nodes in (**a**) the training set with FBP algorithm; (**b**) the validation set with FBP algorithm; (**c**) the training set with IR algorithm; (**d**) the validation set with IR algorithm according to clinical *, radiomics and clinical–radiomics models ^. (* clinical model includes site and nodule size as independent variables; ^ For the FBP algorithm: the clinical–radiomics model includes site, nodule size and radiomics score. For the IR algorithm: the clinical–radiomics model includes site and radiomics score. Nodule size was not included because of overlapping with the radiomic feature Shape_Max3DDiameter).
